# Inflammatory signaling pathways play a role in SYK inhibitor resistant AML

**DOI:** 10.1038/s41598-025-96660-w

**Published:** 2025-04-05

**Authors:** Sarah Tausch, Christina Villinger, Gabriela Alexe, Daniel J. Urban, Min Shen, Dominique Jahn, Jonas Vischedyk, Sebastian Scheich, Hubert Serve, Matthew D. Hall, Kimberly Stegmaier, Thomas Oellerich, Anjali Cremer

**Affiliations:** 1 Department of Medicine, Hematology/Oncology, University Hospital Frankfurt, Goethe University, Theodor-Stern Kai 7, 60594 Frankfurt am Main, Germany; 2 Frankfurt Cancer Institute (FCI), Frankfurt am Main, Germany; 3 Department of Pediatric Oncology, Harvard Medical School, Dana-Farber Cancer Institute and Boston Children’s Hospital, Boston, MA USA; 4 The Broad Institute of MIT and Harvard, Cambridge, MA USA; 5 Division of Preclinical Innovation, National Center for Advancing Translational Sciences, National Institutes of Health, Rockville, MD USA; 6 German Cancer Consortium (DKTK) and German Cancer Research Center (DKFZ), Heidelberg, Frankfurt am Main, Germany

**Keywords:** Acute myeloid leukemia cells, Resistance, Inflammatory pathways, Glucocorticoids, Acute myeloid leukaemia, High-throughput screening

## Abstract

Trials have shown promising clinical activity of the selective SYK inhibitor entospletinib in patients with high expressing *HOXA9/MEIS1* acute leukemias. As the development of resistance mechanisms is a common problem in the use of targeted drugs, we performed a chemical library screen to identify drug sensitivities in SYK inhibitor resistant AML cells. We identified that SYK inhibitor resistant cells displayed an increased sensitivity to glucocorticoids. Glucocorticoids are potent immunosuppressants which work in part by inhibiting the transcription of cytokine genes. RNA sequencing of entospletinib resistant cells revealed a strong enrichment of inflammatory response and TNFα signaling via NF-κB gene sets in comparison to naive cells. Naive AML cells treated with entospletinib showed a strong downregulation of the same gene sets which were upregulated in the resistant state. Our data suggest that inflammatory signaling pathways play a role in entospletinib resistant AML cells.

**Supplementary Information:**

The online version contains supplementary material available at 10.1038/s41598-025-96660-w.

## Introduction

Acute myeloid leukemia (AML) is a heterogenous disease that arises from uncontrolled proliferation of clonal hematopoietic cells^1^. For patients with certain gene expression signatures, e.g. high *HOXA9* and *MEIS1* expressing leukemias outcome is still poor, with a 5-year overall survival of less than 25%^2^. This gene expression signature can be found in leukemias harboring rearrangements of the gene lysine methyltransferase 2A (*KMT2A*), previously known as mixed-lineage leukaemia (*MLL*)^3^ or mutations in *NPM1*^4^. Recently, Menin-Inhibitors have been evaluated in clinical trials and show increased remission rates in patients with *MLL*r acute leukemia^5^. However, not all patients respond. Hence, new targeted therapies for this difficult to treat patient subgroup have to be evaluated. Spleen tyrosine kinase (SYK) has been identified as a therapeutic target in AML^6^, especially in *HOXA9* and *MEIS1* high expressing leukemias^3^. SYK is rarely mutated^7,8^ and is activated by integrin and Fc receptor signaling in AML^6,9^. High SYK activation in the bone marrow of patients with AML has also been associated with poor therapeutic outcomes^10^. The first clinical trials testing SYK inhibitors in patients with AML showed promising results for *HOXA9* and *MEIS1* high expressing leukemias^11^. One of the main challenges of targeted therapies is the development of acquired resistance. One common mechanism of acquired resistance is the activation of important signaling hubs in the cell as a compensatory mechanism to the inhibited drug target^12^. We have previously identified activation of RAS/MAPK/ERK signaling as a major resistance mechanism to SYK inhibition in AML^13^.

While immune modulating drugs have revolutionized treatment regimens in acute lymphoblastic leukemia (ALL)^14^, the use of these drugs in patients with AML has not been established. There is increasing evidence that the activation of pro-inflammatory pathways plays an important role in AML disease progression and resistance to chemotherapy. Interferon-γ response and inflammatory response related mRNA profiles predicted therapeutic resistance to standard “7 + 3” chemotherapy in patients with AML^15^. Analysis of longitudinal bone marrow samples from patients with AML from primary diagnosis to relapse or in cases of primary resistance confirmed these results, indicating an association of upregulated tumor-promoting inflammatory genes with a short event-free survival, as well as relapse in these patients^16^. Activated inflammatory pathways in leukemia cells can even be exploited as “self-directed immunotherapy,” leading to leukemic blast cell death, thus underscoring the complexity and importance of inflammation in AML^17^. The role of inflammation in response to SYK targeted therapies in AML has not been studied to date. In this study, we set out to characterize resistance mechanisms to SYK inhibitors and the potential link between SYK signaling and activation of inflammatory pathways in more detail.

## Results

### Cells with acquired SYK inhibitor resistance show increased sensitivity to glucocorticoids

**Fig. 1:**
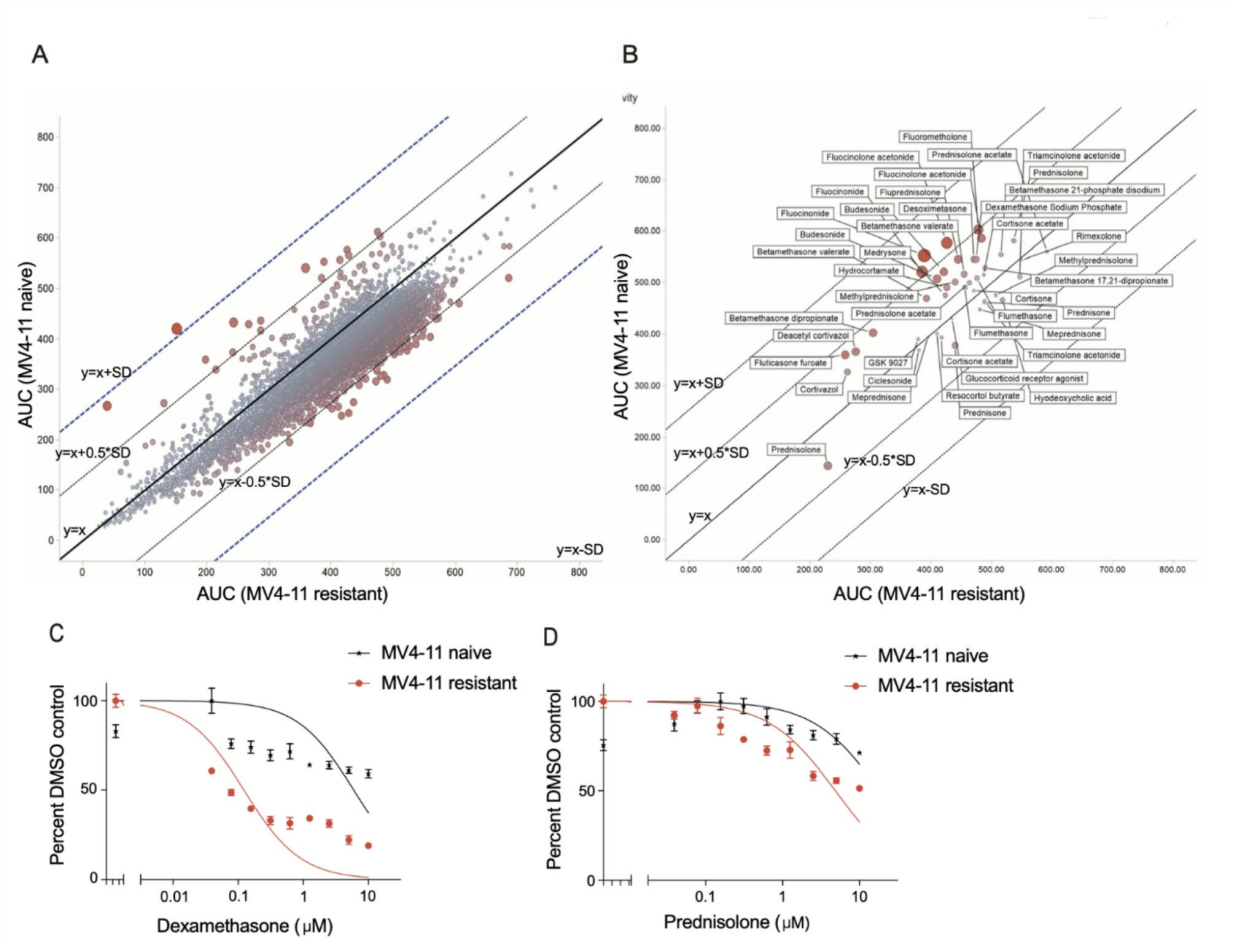
(**A**) Comparative analysis of drug sensitivity between MV4-11 naive and SYKi-resistant cell lines. Scatter plot showing the correlation of AUC (Area Under the dose-response Curve) values for all compounds from the NCATS screening library tested in MV4-11 naive versus resistant cell lines. Each point represents an individual compound, with color intensity and size reflecting potency and differential response (where a stronger red represents greater differential killing between the two cell lines). The black diagonal line represents y = x (equal response in both cell lines), while additional solid and dashed lines indicate ± 0.5 standard deviations (SD) and ± SD from the diagonal. Lower AUC values indicate more potent and efficacious compounds. (**B**) Annotated version of the scatter plot displaying all tested glucocorticoid compounds with differences in AUC between the naive and resistant cell lines. Data points are labeled with the drug/compound names, with those above or below the y = x line indicating potential resistance or hypersensitivity in the resistant MV4-11 cells. Viability analysis using an ATP-based luminescent assay with increasing concentrations of dexamethasone (**C**) and prednisolone (**D**) in MV4-11 naive and SYKi resistant cells.

First, we set out to systematically identify drugs to which entospletinib-resistant AML cells have an increased sensitivity. We screened SYKi resistant MV4-11 cells, which were previously generated by our lab and not found to possess any new pathogenic mutations^13^, against the Mechanism Interrogation PlatE (MIPE)^18^ library of ~ 1900 oncology focused compounds (Fig. 1A). Entospletinib resistant cells showed an increased sensitivity towards glucocorticoids (e.g., betamethasone dipropionate and budesonide) (Fig. 1B). Additionally, entospletinib resistant cells showed an increased sensitivity towards smac mimetics in our drug library screen. However, in this manuscript we focus on glucocorticoids, as the observed underlying mechanisms based on our preliminary data are most likely independent from each other. Glucocorticoids are potent repressors of many genes involved in inflammatory and immune responses, including cytokines and chemokines^19^. After treatment of resistant and parental cells with dexamethasone, we detected a shift in sensitivity in comparison to the naive state (IC_50_ MV4-11 naive: 11.9 µmol/l; MV4-11 resistant: 0.2 µmol/l) (Fig. 1C). We observed a similar but more modest effect upon prednisolone treatment (IC_50_ MV4-11 naive: 18.5 µmol/l; MV4-11 resistant: 4.8 µmol/l) (Fig. 1D). The shift in sensitivity to dexamethasone could also be observed in naive and resistant MOLM14 cells (IC_50_ MOLM14 naive: 28.08 µmol/l; MOLM14 resistant: 2.4 µmol/l) (Supplementary Fig. 1A).

**Fig. 2:**
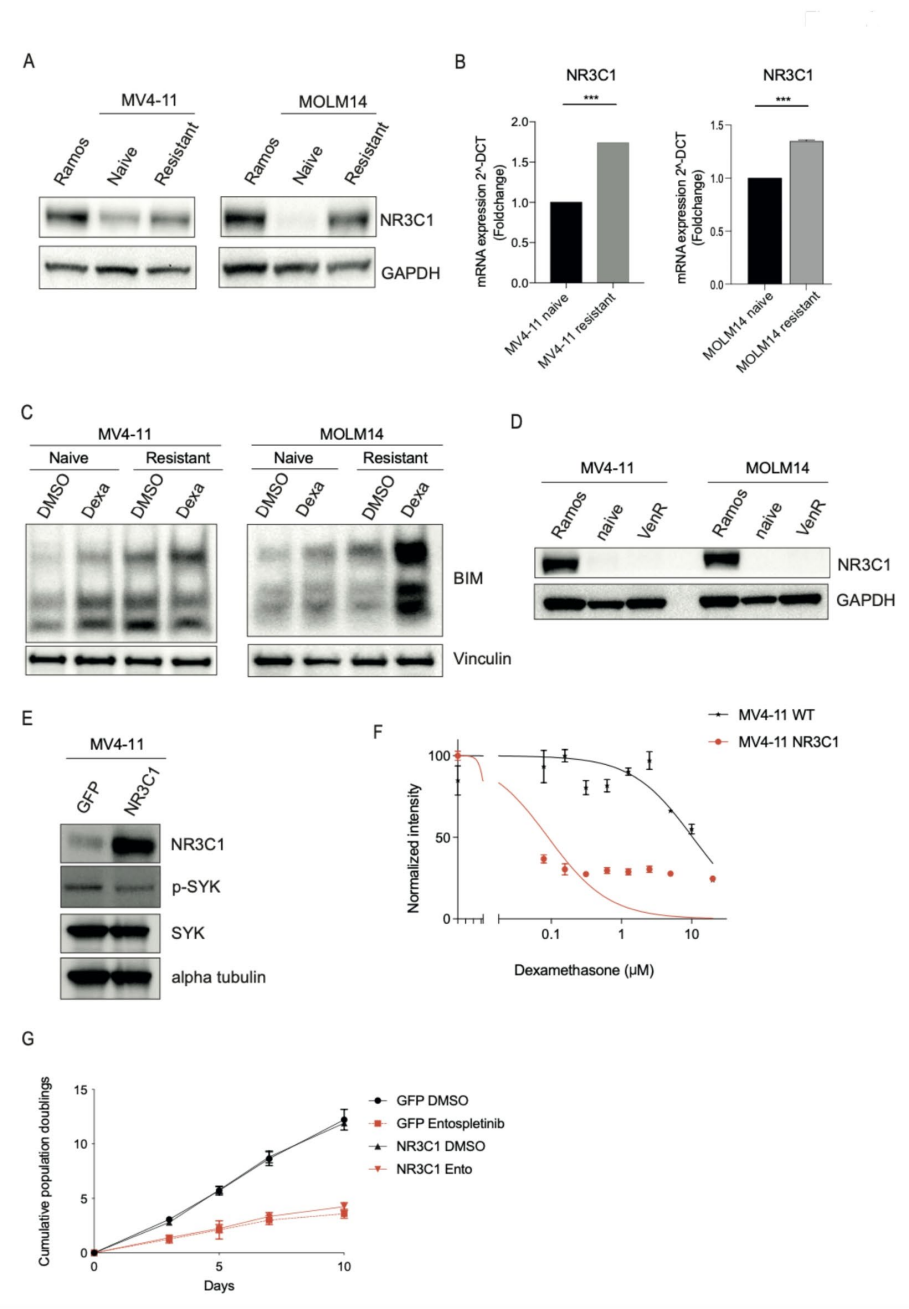
(**A**) Immunoblot showing NR3C1 overexpression in MV4-11 resistant cells. Burkitt lymphoma cell line Ramos, was used as a positive control for NR3C1 expression. GAPDH was used as a loading control. Representative images of three independent replicates are shown. (**B**) Confirmation of NR3C1 mRNA fold-change upregulation in resistant versus naive MV4-11 cells by qpCR (***P ≤ 0.001, Mann-Whitney test). (**C**) Immunoblot showing upregulation of BIM in dexamethasone treated, entospletinib resistant MV4-11 cells. Vinculin was used as a loading control. Representative images of two independent replicates are shown. (**D**) Immunoblot showing NR3C1 expression in naive and venetoclax resistant MV4-11 cells in comparison to Ramos control cells. GAPDH was used as a loading control. Representative images of three independent replicates are shown. (**E**) Immunoblot confirming overexpression of NR3C1 ORF in MV4-11 cells, as well as SYK expression. Alpha tubulin was used as a loading control. Original Blots are presented in Supplementary Fig. 3. (**F**) Viability analysis using an ATP-based luminescent assay with increasing concentrations of dexamethasone in NR3C1 ORF expressing MV4-11 cells. (**G**) Long-term viability assay in MV4-11 cells overexpressing NR3C1 ORF and treated with vehicle or 3 µmol/L entospletinib (Ento).

An increased sensitivity to glucocorticoids has been previously reported in AML cells with acquired resistance to cytarabine and FLT3 inhibitors, as well as in patient AML cells^20,21^, via upregulation of the glucocorticoid receptor encoded by *NR3C1*. A similar mechanism could explain the shift of glucocorticoid sensitivity in the SYKi resistant cells. Indeed, NR3C1 protein (Fig. 2A) and transcript levels were increased (Fig. 2B), as evaluated by immunoblot or qPCR, respectively, in the SYKi resistant state compared to control in both cell lines. Next, we evaluated the effect of treatment of SYKi resistant cells with dexamethasone versus a DMSO control. We could detect a greater upregulation of BIM expression in the resistant cells in comparison to naive cells as detected by immunoblot (Fig. 2C). Upregulation of the pro-apoptotic BH3-only protein BIM induces apoptosis and is a marker for glucocorticoid sensitive cells^22^, suggesting increased apoptosis of SYKi resistant cells after dexamethasone treatment.

To evaluate whether upregulation of *NR3C1* is a non-specific phenomenon in MV4-11 cell lines subjected to long-term culture under continuous drug exposure, protein levels of NR3C1 were evaluated in MV4-11 and MOLM14 cell lines with acquired resistance to venetoclax. Venetoclax resistant cell lines showed no NR3C1 upregulation (Fig. 2D).

In order to determine whether upregulation of *NR3C1* alone confers resistance to SYK inhibitors in AML cell lines, *NR3C1* was overexpressed in wildtype MV4-11 cells (Fig. 2E). NR3C1 overexpression did not have an impact on total SYK protein expression levels in comparison to the control cells, however we detected a slight decrease in phospho SYK expression (Fig. 2E). As expected, overexpression of *NR3C1*, lead to an increased sensitivity to dexamethasone treatment (Fig. 2F). Subsequently *NR3C1* overexpressing cells were treated with entospletinib for 14 days. Overexpression of the glucocorticoid receptor alone was not able to confer resistance to SYK inhibition in AML cells (Fig. 2G), indicating that observed upregulation of *NR3C1* alone is not driving entospletinib resistance.

### SYK Inhibition leads to reduced NF-κB response after TNFα stimulation

SYK is known to be a mediator of immunoreceptor signaling in B-cells. After B-cell receptor engagement, SYK is recruited and activated, mediating downstream signal transduction towards NF-κB^23,24^. There is evidence that SYK and NF-κB signaling are also connected in AML^25^. After *MLL1(KMT2A); MLL2(KMT2D)* knockout in *MLL-AF9* leukemia cells, RNAseq analysis revealed a reduction in the components of three major pathways, namely NF-κB, integrin beta-3 (in which SYK is an intracellular signaling component^26^) and interleukin-3 (IL-3)^25^. Interestingly, *IL3RA* transcript was also confirmed to be reduced in *MLL1/MLL2* double knockout leukemia cells^25^. MLL2-deficient leukemia cells could be partially rescued by culturing cells in the presence of high (10 ng/mL) IL-3, whereas the double knockout cells could only be slightly rescued^25^. It was previously described that treatment involving the combination of the IkB kinase inhibitor VII, targeting NF-κB signaling, a SYK inhibitor and reduced IL-3 had a synergistic effect in leukemia cells^25^.

**Fig. 3:**
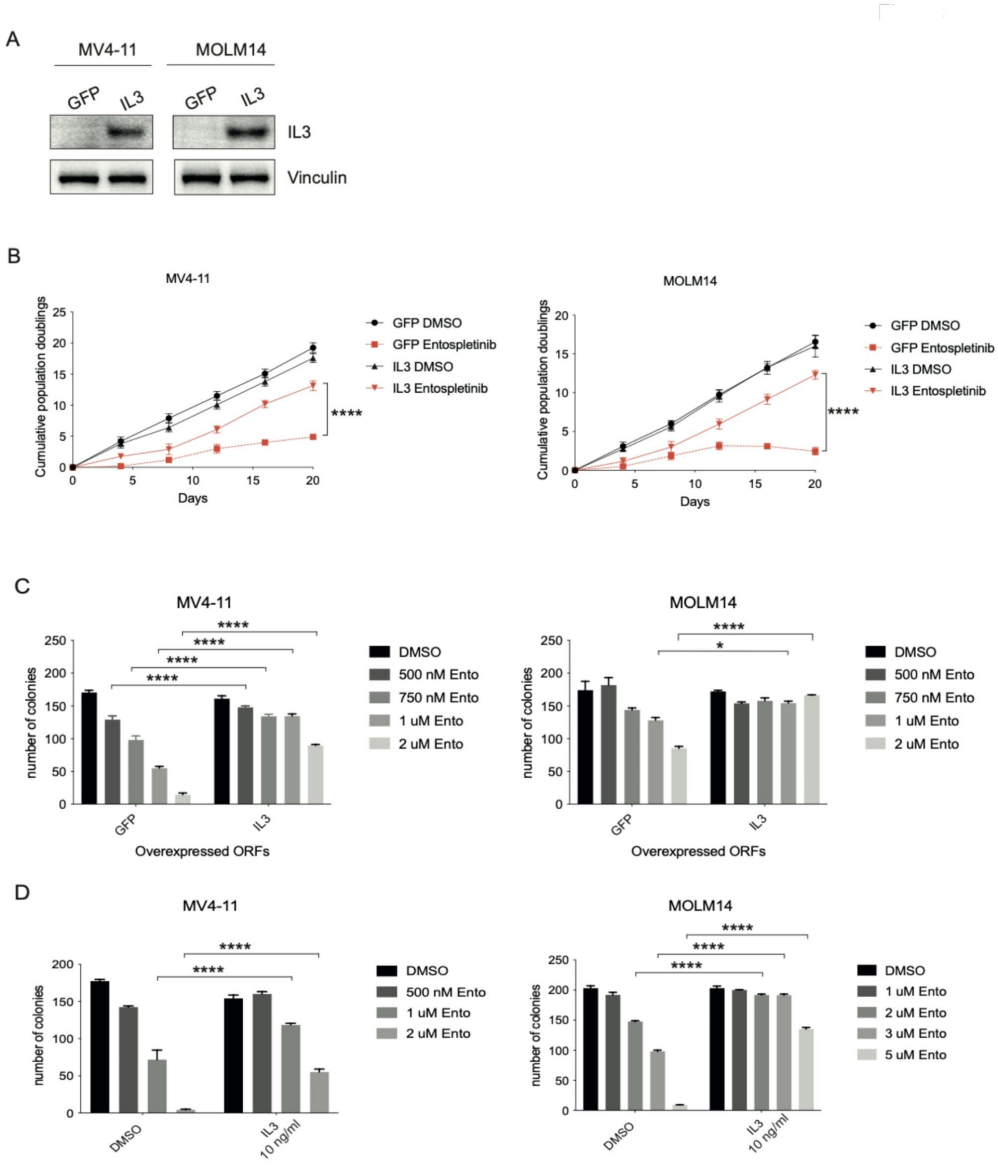
(**A**) Immunoblot confirming overexpression of IL3 ORF in MV4-11 cells. Vinculin was used as a loading control. Representative images of three independent replicates are shown. Original Blots are presented in Supplementary Fig. 4. (**B**) Long-term viability assay in MV4-11 (left) and MOLM14 (right) cells overexpressing the indicated ORFs and treated with vehicle or 3 µmol/L entospletinib (Ento) (****P ≤ 0.0001, two-way ANOVA test, multiple comparisons) (**C**) Colony forming assay in MV4-11 (left) and MOLM14 (right) cells overexpressing the indicated ORFs and treated with increasing concentrations of entospletinib (Ento). (*P ≤ 0.05, ****P ≤ 0.0001, two-way ANOVA test, multiple comparisons) (**D**) Colony forming assay with vehicle or IL3 addition (10 ng/ml) to methylcellulose in MV4-11 (left) and MOLM14 (right) cells treated with increasing concentrations of entospletinib (****P ≤ 0.0001, two-way ANOVA test, multiple comparisons).

In line with these observations, a genome-scale, lentiviral ORF library screen, previously performed by our lab, identified IL-3 overexpression as a major resistance mechanism to SYK inhibition in AML^13^. Overexpression of the *IL3* gene in the screen conveyed resistance to treatment with the SYK inhibitor entospletinib in the two *FLT3* mutant AML cell lines, MOLM14 and MV4-11. In order to validate our findings, we re-expressed an *IL3* ORF in MV4-11 and MOLM14 cells. Overexpression was confirmed by using antibodies specific to IL-3 (Fig. 3A). Next, these cells were treated with either entospletinib or DMSO as a control to determine whether the overexpressed genes could rescue the effects of SYK inhibitor treatment. Overexpression of *IL3* promoted resistance to entospletinib-mediated cell death (Fig. 3B), confirming the ORF screen data. In addition, we observed that cells overexpressing *IL3* had increased colony forming potential in comparison to control cells treated with entospletinib (Fig. 3C). This finding was confirmed by adding IL-3 to methylcellulose in wildtype MV4-11 cells and MOLM14 cells (Fig. 3D). These findings are in line with the previous observation that IL-3 can rescue the effects of *MLL1* and *MLL1/MLL2* knockout in leukemia cells^25^, which is consistent given the fact that SYK inhibition results in the downregulation of the same gene expression signature as *HOXA9/MEIS1*-driven leukemia^3^.

**Fig. 4:**
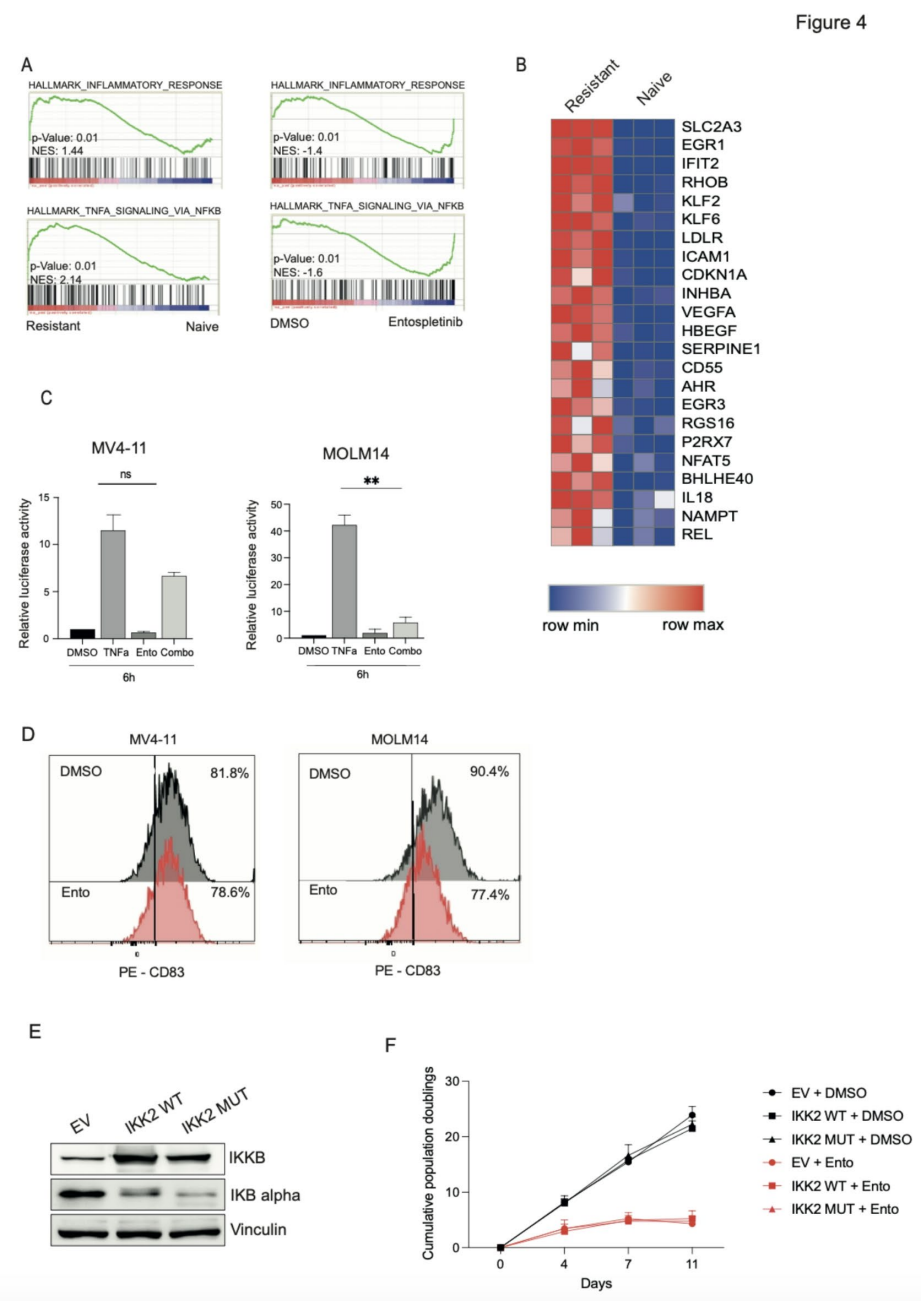
(**A**) GSEA demonstrating enrichment of signatures upregulated in resistant cells in comparison with naive cells, as well as in cells treated with entospletinib (**B**). Shown is a heatmap of leading edge genes that were included in both gene sets “Inflammatory response” and “TNFα signaling via NF-kB” in resistant cells in comparison with naive cells. Significance was assessed based on NES ≥ 1.5, *P* ≤ 0.01. (**C**) Relative luciferase activity in MV4-11 cells harboring a NF-kB luciferase reporter assay after TNFa stimulation or entospletinib treatment or both (combo). DMSO was used as a control. (**D**) CD83 expression measured by FACS in MV4-11 and MOLM14 cells after entospletinib treatment. (**E**) Immunoblot confirming increased IKKB and reduced IKB alpha after overexpression of wildtype or mutant IKK2 in MV4-11 cells. Vinculin was used as a loading control. Representative images of two independent replicates are shown. Original Blots are presented in Supplementary Fig. 4. (**F**) Long-term viability assay in MV4-11 cells overexpressing the indicated wildtype or mutant IKK2 and treated with vehicle or 3 µmol/L entospletinib (Ento).

To investigate which signaling pathways are activated in the entospletinib resistant state, bulk RNA sequencing of entospletinib resistant MV4-11 cells was performed^13^. Here, we observed a strong correlation between the SYKi resistant cell state and inflammatory response (Normalized enrichment score (NES) = 1.44, FDR < 0.001, *P* < 0.01), and TNFα signaling via NF-κB (NES = 2.14, FDR < 0.01, *P* < 0.01) in GSEA (Fig. 4A, left). Leading edge genes that were upregulated in both pathways in SYKi resistant cells included known NF-κB target genes such as *ICAM1*,* REL*, and *EGR1* (Fig. 4B*)*. This data suggests that the upregulation of NF-κB signaling might play a role in conferring resistance to SYK inhibition in AML. Subsequently, we investigated whether inhibition of SYK by entospletinib treatment of naive AML cells led to a downregulation of inflammatory pathways in AML cells via RNAseq analysis^13^. GSEA on this data revealed a strong correlation between entospletinib treatment and the downregulation of the gene sets “Inflammatory response” (NES= -1.4, FDR < 0.001, *P* < 0.01) and “TNFα signaling via NF-κB” (NES= − 1.6, FDR < 0.01, *P* < 0.01) (Fig. 4A, right). These gene sets were also found to be upregulated in the SYKi resistant state (Fig. 4A, left).

NF-κB represents a family of transcription factors that are normally kept inactive in the cytoplasm through interaction with inhibitory molecules of the IκB family^27^. Upon activation via inflammatory cytokines or other types of cell stress, IκB molecules become subject to proteasomal degradation. Consequently, NF-κB translocates to the nucleus and activates the transcription of genes that participate in inflammatory response, growth control, and protection against apoptosis^27^. One of the most potent activators of NF-κB is TNFα, and it has been reported that TNFα-induced NF-κB activation is mediated via SYK activation in a T-cell leukemia cell line^28^. Based on our RNAseq data we hypothesized that SYK inhibition leads to reduced activation of NF-κB signaling in AML. To investigate this, we first established a NF-κB luciferase reporter assay^29^ in MV4-11 and MOLM14 cells. When cells were treated with TNFα for 3 h (h) at a concentration of 20 ng/ml, we could detect an upregulation of NF-κB signaling (Fig. 4C). After combining TNFα treatment with entospletinib treatment, a decreased response to TNFα in MV4-11 (not significant) and MOLM14 (**p = 0.0068) cells was observed (Fig. 4D). Analysis of the expression of CD83, a known NF-κB target gene, in wildtype MOLM14 cells treated with SYKi revealed a decrease of CD83 expression in comparison to the control (Fig. 4D, right); however, we could not observe a decrease in CD83 expression in MV4-11 cells (Fig. 4D, left).

We next investigated whether constitutively active NF-κB signaling alone can mediate resistance to entospletinib in wildtype MV4-11 cells. Therefore, wildtype IKK-2 and a double mutant form of IKK-2 (S177E, S181E)^30^ leading to constitutive active IKK-2 was overexpressed by lentivirus in MV4-11 cells. Overexpression of IKK-2 and the double mutant was confirmed by immunoblot (Fig. 4E). As expected, overexpression of IKK-2 lead to increased degradation of IKB alpha as detected by immunoblot, confirming activation of NF-κB^27^. However, when we treated the cells with entospletinib or DMSO as a control we could not detect a difference in doubling times of the cells (Fig. 4F), indicating that constitutively activated NF-κB signaling by overexpression of IKK-2 alone does not convey resistance to SYK inhibition in wildtype MV4-11 cells. These data suggest that prior continuous exposure with a SYK inhibitor facilitates activation of NF-κB signaling in AML cells.

## Discussion

AML is a heterogenous disease with an estimated 5-year OS of 30%. Prognosis differs greatly between age groups, reaching 50% in younger patients but is less than 10% in patients older than age of 60^1^. Due to the approval of 11 new drugs or combinations since 2017 [31–35] (e.g. midostaurin, gilteritinib, ivosidenib, venetoclax) outcome of patients with AML will most likely improve in the near future. It is known that one of the greatest challenges of targeted therapies is the emergence of intrinsic or acquired resistance to these drugs. Understanding the underlying mechanisms of resistance (e.g. mutations, rewiring of signaling hubs) is important to propose potential strategies to overcome them.

Inflammation is a critical component of tumor progression. Many cancers arise from sites of infection, chronic irritation and inflammation^31^. NF-*κ*B is a transcription factor that can be activated by a variety of cytokines and plays a key role in inflammatory processes. In transformed cancer cells, it is usually kept active and can regulate the cell cycle and apoptosis process by activating genes encoding related proteins, thereby inducing survival and promoting cancer progression^32^. The role of activation of inflammatory signaling pathways in drug resistance in AML has not been studied in detail yet.

This study shows that SYKi resistant cells display an upregulation of inflammatory pathways by RNAseq analysis. Chemical SYK inhibition in SYKi naive AML cells results in a dampened TNFa- induced NF-kB activation, implying that the pathway is involved in SYK signaling in AML cells. Our data provide evidence that upregulation of the glucocorticoid receptor is not only restricted to the selective pressure induced by cytarabine or FMS-like tyrosine kinase III (FLT3) inhibitor resistance in AML as reported previously^20,21^, but can be also observed in cells with acquired resistance to SYK inhibition, leading to an increased sensitivity to glucocorticoids in two SYKi resistant AML cell lines. The preemptive identification of resistance mechanisms to targeted therapies have the potential to better inform clinical trials and improve outcomes for patients in the future.

Our experiments were conducted in vitro using AML cell lines, which is a limitation of this study, as cell line models are not able to take the complex in vivo bone marrow microenvironment of AML patients into account, which is important in regulation and response to cytokines and chemokines secreted by immune cells^33^. However, to study acquired resistance mechanisms that are not conveyed by genetic mutations it is still the best model there is, as patient cells can not be maintained for a longer period of time in cell culture without the addition of various cytokines to cell culture media that alter signaling pathways in itself.

In summary, our study demonstrates that inflammatory signaling pathways play a role in conferring resistance to entospletinib, which can be reversed in part by treatment with anti-inflammatory drugs. However, whether observed effects are direct or indirect, as well as the exact underlying mechanism remains elusive and will require further investigation in the future.

## Methods

### Cell lines

MV4-11 and MOLM14 cells were previously provided by Scott Armstrong. Both cell lines were maintained in RPMI 1640 (Cellgro) supplemented with 1% penicillin/streptomycin (PS; Cellgro) and 10% FBS (Sigma-Aldrich) at 37 °C with 5% CO_2_. Ramos cells were provided by Thomas Oellerich and were maintained in RPMI 1640 (Cellgro) supplemented with 1% penicillin/streptomycin (PS; Cellgro) and 20% FBS (Sigma-Aldrich) at 37 °C with 5% CO_2_. The 293T cells were maintained in Dulbecco’s modified Eagle’s medium (Invitrogen) supplemented with 10% FBS (Invitrogen) and 1% PS. All cell lines were tested negative for *Mycoplasma* and were authenticated using short tandem repeat (STR) profiling.

*Generation of entospletinib resistant cells* To generate cells that were resistant to SYK inhibition, MV4-11 were treated for 5 months with gradually increasing concentrations of entospletinib (500 nM–5 µM). Cells were considered entospletinib-resistant when they were able to maintain a 90–100% viability in the presence of entospletinib at a 10-fold higher concentration than the IC_50_.

### Chemicals

Compounds used for high-throughput screening were sourced from the NCATS compound library. All compounds for in vitro experiments were obtained from Selleck.

### Lentiviral/Retrovirus production and transduction

Lentivirus was generated by transfecting HEK-293T cells with the indicated vectors and the packaging plasmids, delta8.9 and VSVG, following the X-tremeGENE HP DNA Transfection Reagent protocol (Roche). AML cells were infected with 2 mL of virus and 8 µg/mL polybrene.

Retrovirus was generated by using the packaging plasmids, ψEco and RV. Primary murine bone marrow cells were infected by spin-infection in a centrifuge with 50 µl of virus and 8 µg/mL polybrene in a 96-well plate, at 1500 rpm for 2 h at room temperature (RT). Cells were selected with puromycin containing media 48 h after infection.

### Validation of ORF hits

Lentiviral infected cells expressing candidate ORF hits from the primary screen were seeded in TC25 flasks in technical duplicate and were treated with DMSO or 3 µM entospletinib. Cumulative population doublings were calculated by manually counting cells every 3–4 days for a total of 21 days.

### RNA-sequencing

RNA was extracted from cells with the RNeasy Kit and on-column DNA digestion (Qiagen).

For RNA-sequencing of MV4-11 cells, polyA mRNA was isolated and libraries were prepared using the TruSeq Stranded mRNA Kit (Illumina) according to the manufacturer’s protocol. All samples were sequenced on a NextSeq500 instrument with single-end 75 bp reads to a depth of 30-50 M reads/sample. RNAseq was performed after SYKi resistant cells were cultured for 24 h in drug free media.

### Plasmids

*IKK-2* WT and *IKK-2* S177E S181E were a gift from Anjana Rao (Addgene plasmid #11103 and #11105). pHAGE NFkB-TA-LUC-UBC-GFP-W was a gift from Darrell Kotton (Addgene plasmid #49343). The NF-κB reporter assay contains four tandem copies of the NF-κB consensus sequence located upstream of the minimal TA promoter (Tap) the TAT box of the HSV-TK promoter, downstream from the Tap is the firefly luciferase reporter gene as well as a ubiquitously expressed GFP reporter under the control of the constitutive mammalian ubiquitin C (UBC) promoter^29^.

### Genome-scale ORF screens

The ORFeome barcoded library contains 17,255 barcoded ORFs overexpressing 10,135 distinct human genes with at least 99% nucleotide and protein match. Screening-scale infections of the ORFeome library were performed with cells sufficient to achieve a representation of at least 1000 cells per ORF (~ 2 × 10^7^ surviving cells containing 17,255 ORFs). Infections were performed with the pre-determined virus volume in the 12-well format, as the viral titration described above, and pooled 24 h post-infection. Approximately 24 h after infection, all wells within a replicate were pooled and 48 h after infection, cells were selected with puromycin. After selection was completed, 3 × 10^7^ cells were divided into drug treated (3 µM entospletinib for MV4-11 and MOLM14) and vehicle treated arms. Cells were passaged in drug or fresh media containing drug every 3–4 days, and throughout the screen an average representation of 1000 cells per ORF construct was maintained. Cells were harvested 21 days after initiation of treatment. For both ORF screens, genomic DNA (gDNA) was isolated using Maxi (2 × 10^7^–1 × 10^8^ cells) or Midi (5 × 10^6^–3 × 10^7^ cells) kits according to the manufacturer’s protocol (Qiagen). PCR and sequencing were performed as previously described^34^.

### RT-PCR

The expression of *NR3C1* was quantified utilizing RT-PCR with a taqman probe specific for *NR3C1* (ThermoFisher Scientific, Assay-ID Hs00353740_m1).

### Western blotting

Proteins were extracted using Lysis Buffer (Cell Signaling Technology) supplemented with Complete, Ethylenediaminetetraacetic acid (EDTA)-free Protease Inhibitor Cocktail (Roche Diagnostics) and Phosphatase Inhibitor Cocktail (Roche Diagnostics). Protein samples were separated by SDS-PAGE and subsequently transferred to polyvinylidene difluoride (PVDF) membranes, which were blocked in 5% BSA and incubated with primary antibodies against NR3C1 (D6H2L) XP (Cell Signaling Technology, Cat. No. 12041), BIM (Epitomics, Cat. No. 1036-1), IKKβ/IKK2 (D30C6) (Cell Signaling, Cat. No. 8943), IκBα (L35A5) (Cell Signaling Technology, Cat. No. 4814), IL-3 (Santa Cruz, Cat. No. sc-28342), phospho- SYK Y525/Y526 (Cell Signaling Technology, No. 2711), SYK (Santa Cruz Biotechnology, No. sc-1240),GAPDH (D16H11) XP (Cell Signaling Technology, Cat. No. 5174), α-Tubulin (11H10) (Cell Signaling Technology, Cat. No. 2125), or Vinculin (Sigma, Cat. No. V9131). Membranes were washed in TBS-T and incubated with the appropriate horseradish peroxidase-conjugated secondary antibodies. Signal was detected by enhanced chemi-luminescence (ThermoFisher Scientific). Image acquisition was performed using ChemiDoc Imaging System (BIO-RAD). Protein concentration of three or two independent replicates was statistically quantified using ImageJ.

### Cell viability assay

Cells were resuspended at 15,000 cells/mL and seeded at 40 µL/well into 384-well plates. Cells were then treated with a single agent or DMSO as a control and analyzed for cell viability on days 0 and 3 post-treatment using the CellTiter-Glo Luminescent Assay Kit (Promega) according to the manufacturer’s protocol. Luminescence was read on a TECAN reader.

### Long term viability assay

To determine long term viability of cells, 1E6 cells per 10 ml medium were seeded into a T25 culture flask. Cells were treated with 3µM/mL Entospletenib, or DMSO as control. Cell count and viability was determined every 4 days with a TC20 automated cell counter (Bio-Rad), using these values cumulative population doublings were calculated.

### High-throughput viability screen

A high-throughput screen was conducted in 1536-well white flat bottom plates (Corning). Using a MultiDrop Combi (Thermofisher Scientific), 2 µL of media were added to the plates. Compounds from four drug screening libraries (MIPE 5.0, NPC, NPACT, and Kinase Library) totalling 13,72 compounds were dissolved in DMSO and then added to plates using an acoustic dispenser. Cell lines were seeded into plates at a final density of 500 cells in 5 µL of media per well (MultiDrop Combi). After 72 h of compound incubation, 3 µL of Cell-TiterGlo reagent (Promega) was added to each well. Following a 10 min incubation, luminescence was read using the Viewlux microplate reader (PerkinElmer). Primary screening data is publicly available with PubChem AIDs 1,963,823 (https://pubchem.ncbi.nlm.nih.gov/bioassay/1963823) and 1,963,824 (https://pubchem.ncbi.nlm.nih.gov/bioassay/1963824).

### Quantification and statistical analyses

GSEA v2.1.0, GraphPad PRISM 7, R 3.2.3 and Python 2.7.2 software packages were used to perform the statistical analyses. Statistical tests used are specified in the figure legends. Errors bars represent standard deviation, unless otherwise stated. The threshold for statistical significance is p-value ≤ 0.05, unless otherwise specified.

## Electronic supplementary material

Below is the link to the electronic supplementary material.

Supplementary Material 1

## Data Availability

Chemical drug library screen data is available with PubChem AIDs (https://pubchem.ncbi.nlm.nih.gov/bioassay/1963823) and (https://pubchem.ncbi.nlm.nih.gov/bioassay/1963824). RNA-seq data was deposited at the Gene Expression Omnibus (GEO) under accession number GSE129698.
