# Supplementary material for: Inflammatory signaling pathways play a role in SYK inhibitor resistant AML
Source: Sci Rep. 2025 Apr 5;15:11673. doi: 10.1038/s41598-025-96660-w (PMC11972322; doi:10.1038/s41598-025-96660-w)

Inflammatory signaling pathways play a role in SYK inhibitor resistant AML

Sarah Tausch, Christina, Villinger, Gabriela Alexe, Daniel J. Urban, Min Shen, Dominique Jahn, Jonas Vischedyk, Sebastian Scheich, Hubert Serve, Matthew D. Hall, Kimberly Stegmaier, Thomas Oellerich, Anjali Cremer

A

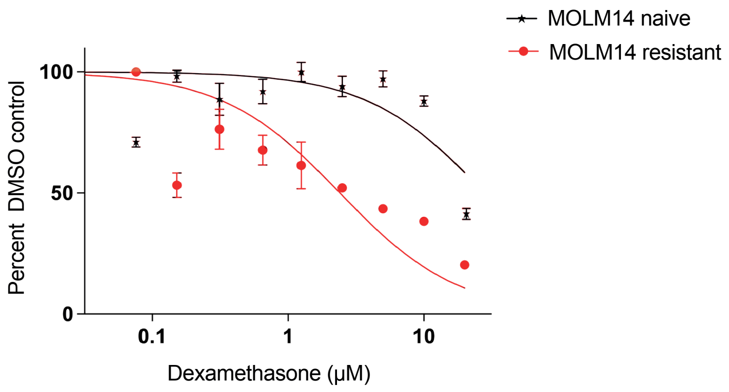

Supplementary Figure 2

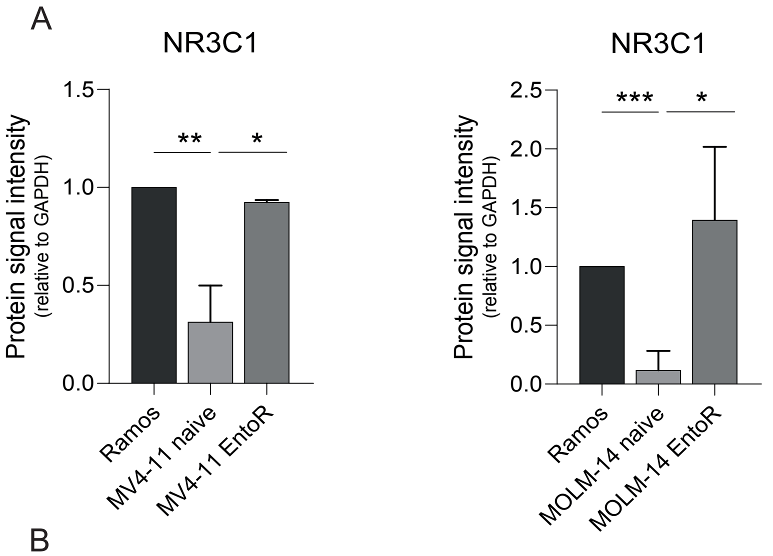

Figure Caption Supplementary Figure 2

A) Quantification NR3C1 - 3 replicates - Fig. 2A
B) Quantification BIM - 2 replicates - Fig. 2C
C) Quantification NR3C1 - 2 replicates - Fig. 2D
D) Quantification NR3C1- 3 replicates - Fig. 2E
E) Quantification IL3 - 3 replicates - Fig. 3A
F) Quantification IKKB and IKB alpha - 2 replicates - Fig. 4E

For statistical analysis of protein quantification ImageJ was used (ns, P ≥ 0.05; *, P ≤ 0.05; **, P ≤ 0.01; ***, P ≤ 0.001; ****, P ≤ 0.0001; t-test)

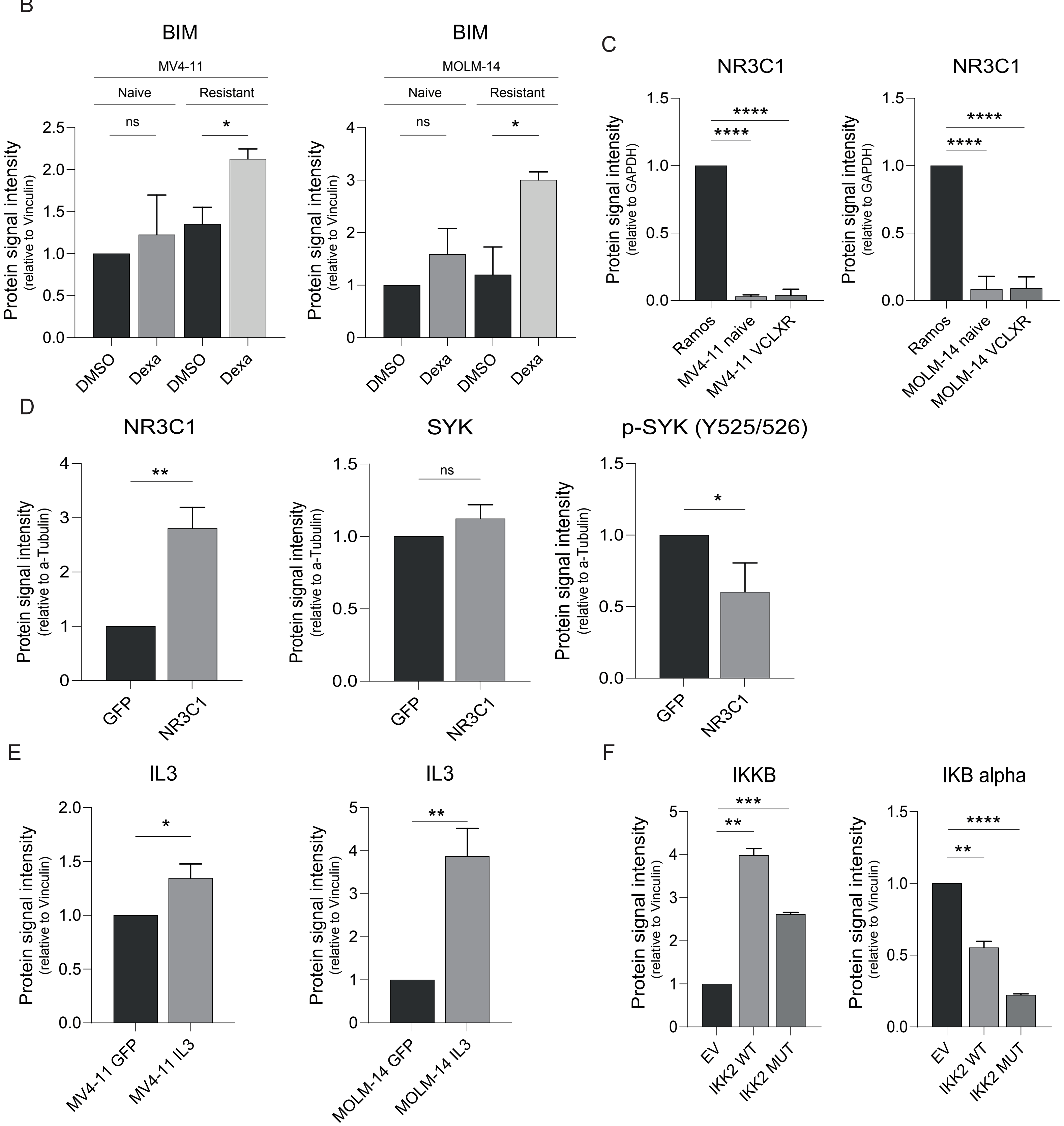

Supplementary Figure 3

A) Immunoblot Fig. 2A
B) Immunoblot Fig. 2C
C) Immunoblot Fig. 2D
D) Immunoblot Fig. 2E

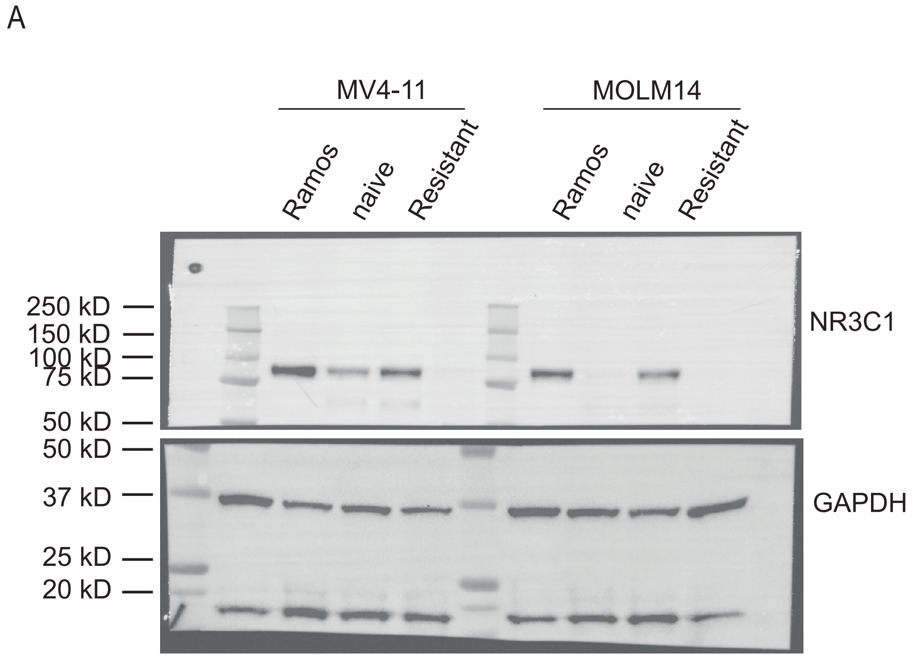

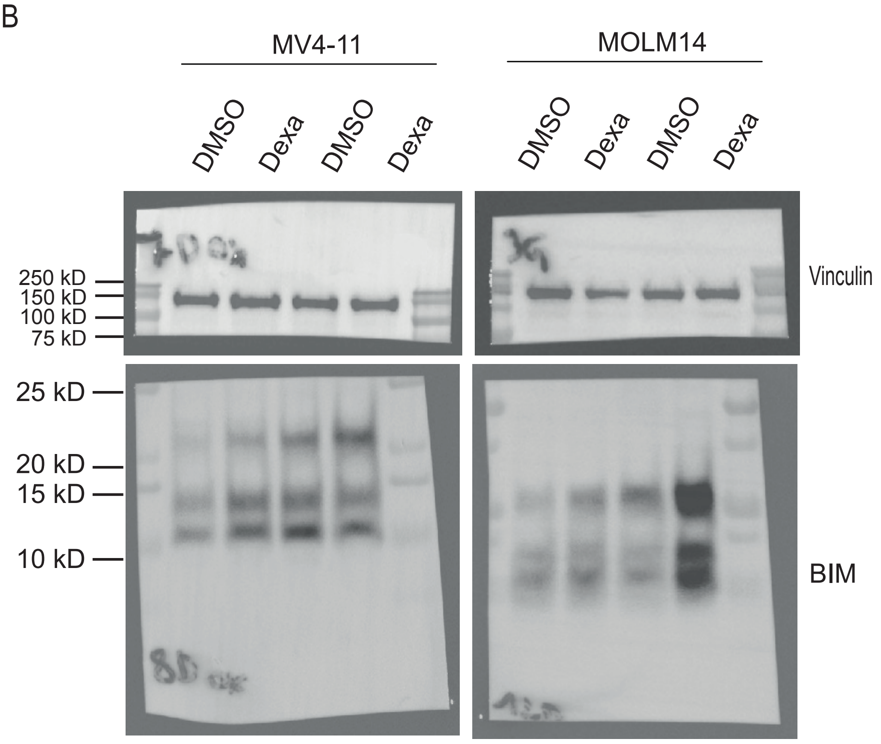

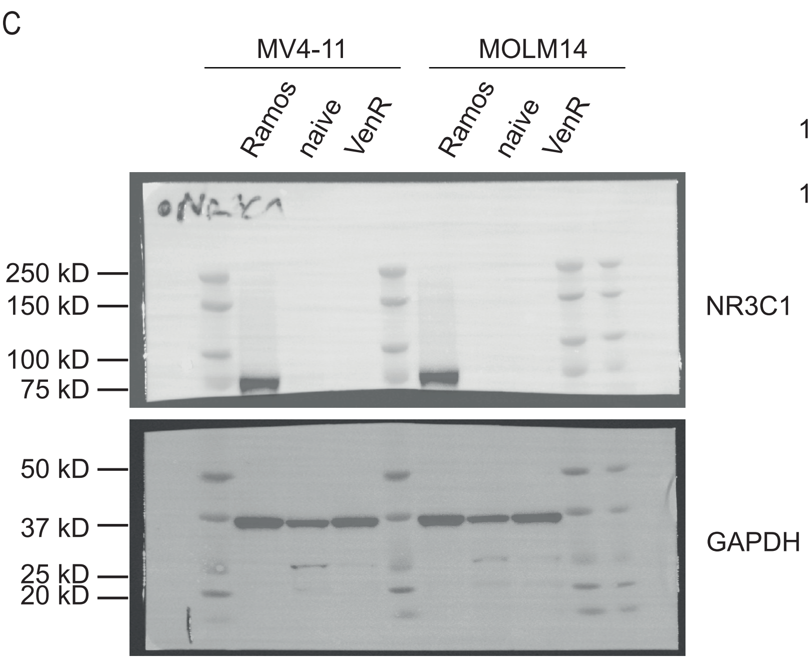

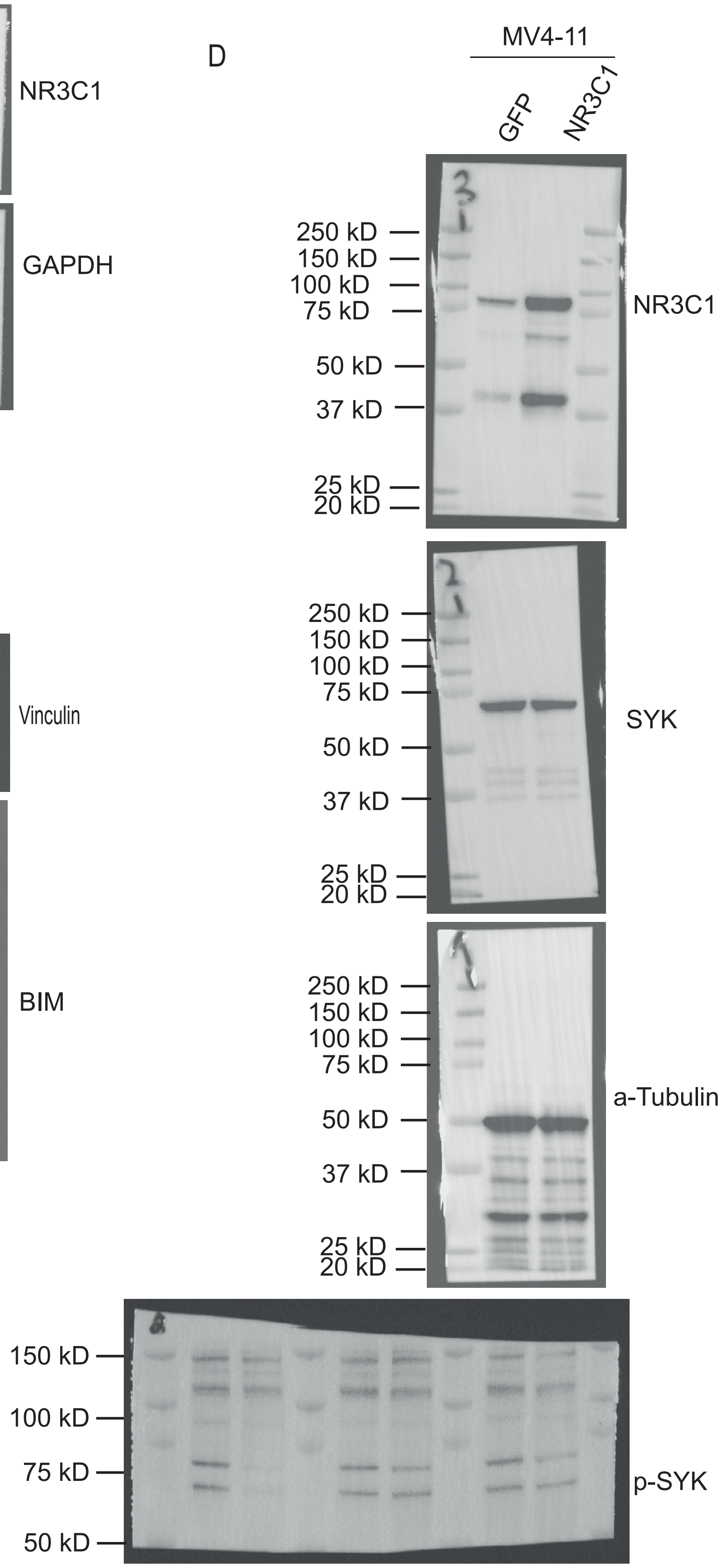

Supplementary Figure 4

A) Immunoblot Fig. 3A
B) Immunoblot Fig. 4E

A

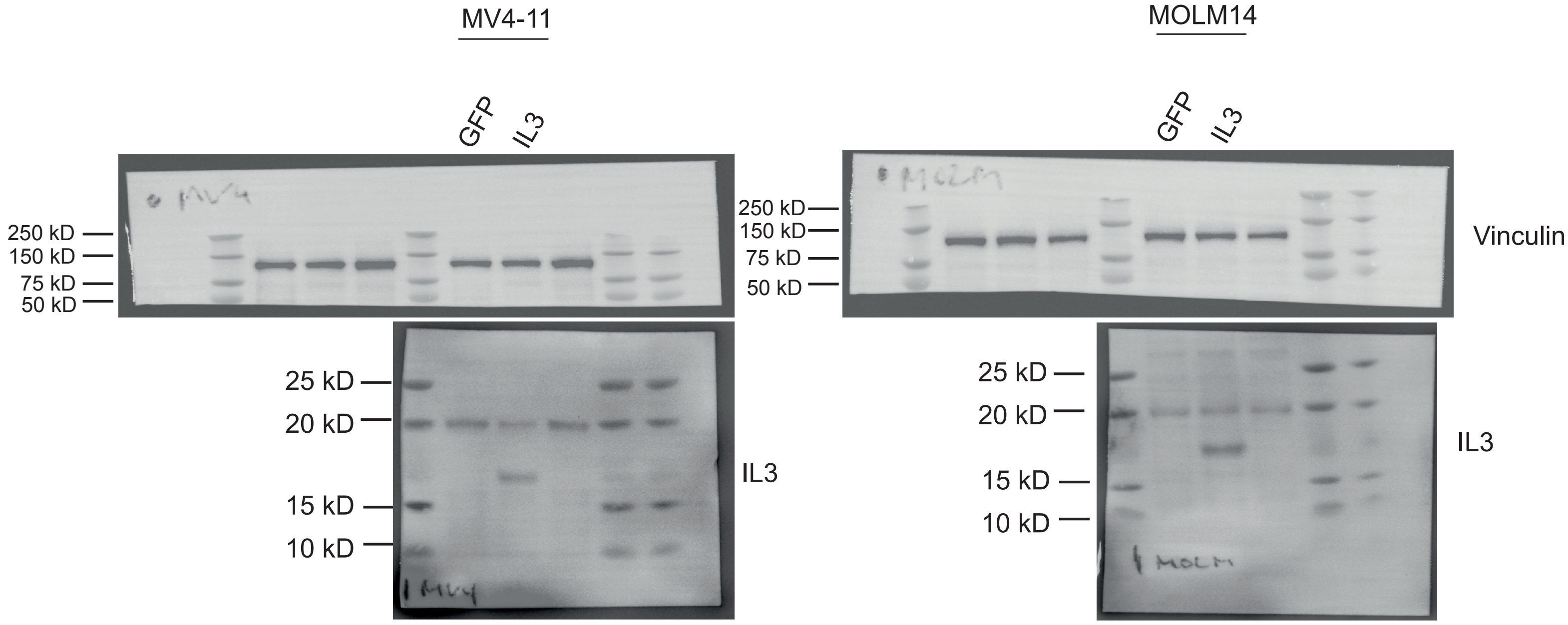

B

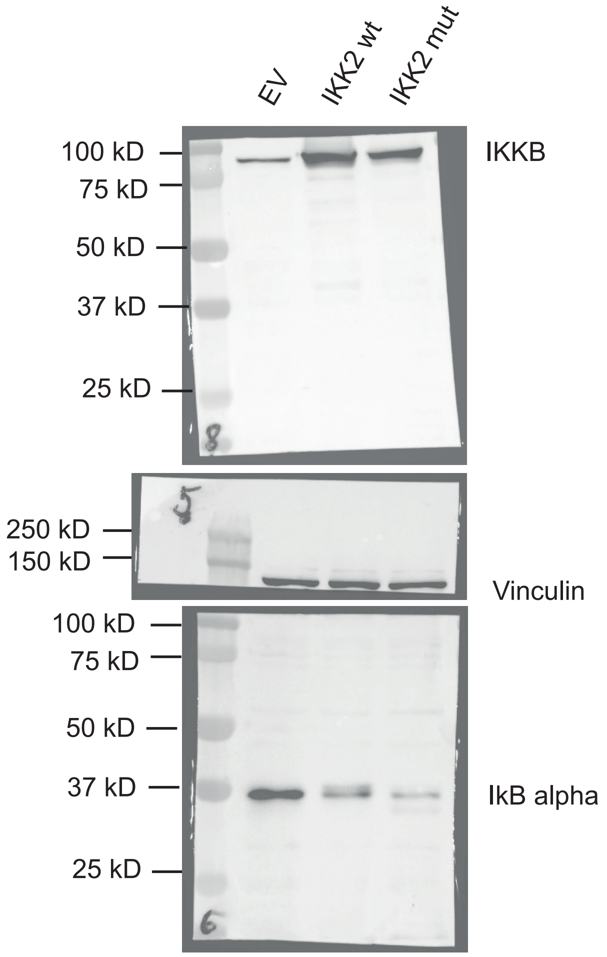

Supplement: Supplementary file 1 — Supplementary Material 1 [file 41598_2025_96660_MOESM1_ESM.pdf]
